# Outcomes of ALK positive lung cancer patients treated with crizotinib or second-generation ALK inhibitor: a monoinstitutional experience

**DOI:** 10.18632/oncotarget.24573

**Published:** 2018-02-26

**Authors:** Elisa De Carlo, Maria Chiara Del Savio, Jerry Polesel, Valentina Da Ros, Eleonora Berto, Sandra Santarossa, Emanuela Chimienti, Lucia Fratino, Alessandra Bearz

**Affiliations:** ^1^ Medical Oncology Department, CRO-IRCCS, Aviano, Italy; ^2^ Epidemiology Unit, CRO-IRCCS, Aviano, Italy

**Keywords:** non-small cell lung cancer, anaplastic lymphoma kinase (ALK), ALK inhibitors, brain metastasis

## Abstract

Rearrangement in the anaplastic lymphoma kinase (ALK) gene is one of the oncogenic drivers in non-small cell lung cancer (NSCLC) patients.

Several ALK inhibitors (ALKis) have been developed and have demonstrated their efficacy, however the best treatment strategy for ALK positive NSCLC patients has yet to be determined. Our retrospective study has investigated the outcome of 40 ALK-rearranged NSCLC patients treated with two different sequential strategies in our Institute; a “classical group”, treated with crizotinib followed by second or third generation ALKis, and the “experimental group”, treated upfront with a second generation ALK inhibitor. The primary endpoints investigated were Progression-free survival (PFS) and intracranial activity. The analysis has revealed a significant improvement in PFS (*p* = 0.050) in the experimental group, furthermore none of these patients developed brain metastasis. There was no statistically significant difference in OS, but all patients in the experimental group were still alive after a median follow up of 15 months. Our retrospective analysis suggests that systemic and intracranial efficacy tends to be better in the experimental group; randomized prospective studies could confirm our observations.

## INTRODUCTION

The echinoderm microtubule-associated protein-like 4 (EML4)-anaplastic lymphoma kinase (ALK) fusion gene was identified for the first time in 2007 in a 62 year-old male patient with lung adenocarcinoma, ALK-rearrangements serve as a key oncogenic driver that occur in 3%–7% of patients with NSCLC [1, 2]. NSCLC tumors harboring the EML4-ALK fusion transcript are sensitive to ALK tyrosine kinase inhibitors (ALK-TKIs).

Crizotinib, a first-in-class ALK-TK inhibitor, demonstrated a significant improvement in both PFS and Overall response rate (ORR) over standard chemotherapy in first and second-line setting, in two randomized phase III trials [3, 4]. In the PROFILE-1014 PFS was 10.9 months and RR was 74%, with crizotinib over platinum-based chemotherapy [4]. Based on these results, crizotinib received approval from Food and Drug Administration (FDA) and European Medicines Agency (EMA) for first-line treatment of ALK-positive patients. Most of the patients develop resistence to crizotinib and thus a disease progression within one year of the onset of the therapy [4].

Significant progress in understanding the biology of ALK-positive tumors has been made and the introduction of potent second and third generation ALKis has improved the treatment options for this subgroup of patients.

Different mechanisms of acquired resistance to crizotinib, including secondary ALK mutations (40% of cases), ALK fusion, gene amplification and activation of alternative signaling pathways, have been identified [5–7]. Moreover, ALK-positive NSCLC patients have a high risk of developing brain metastases, as observed in approximately 30% of cases at the time of tumor diagnosis and in 60% of patients during crizotinib treatment [8, 9]. Crizotinib has a poor blood brain barrier (BBB) penetration and a quite high percentage of failure in central nervous system (CNS) has been observed [10–12]. The second and third generation ALKis have been developed with the aim to overcome secondary-resistance ALK mutations and increase the intracranial activity.

Ceritinib, a small ATP-competitive ALK TK inhibitor, approximately 20 times more potent than crizotinib, was granted FDA accelerated approval in April 2014 as well as conditional EMA authorization in May 2015 for the treatment of ALK-positive patients who progressed on or are intolerant to crizotinib due to an ORR of 56% and a median PFS of 6.9 months observed in the cohort of ALK-TKI pre-treated patients enrolled in the ‘ASCEND-1’ trial [13]. The phase III ASCEND-4 and ASCEND-5 trials showed significantly longer PFS and higher ORR of ceritinib over first line and second line chemotherapy in crizotinib naive and pre-treated patients, respectively [14, 15].

Alectinib is a highly potent selective oral ALK TKI, active in crizotinib-naïve and crizotinib-resistant patients, with proven activity against the L1196, the C1156Y, the F1174L, the R1275Q and the G1269A ALK secondary mutation [16]. Since alectinib is not a substrate of P-glycoprotein, it may reach effective therapeutic concentrations in CNS [17–21]. Intracranial ORR of 64%, with a disease control rate of 80% and a median duration of response of 10.8 months were registered in the pooled analysis, including 136 patients with brain lesions, receiving alectinib in the two phase II studies, the NP28761 and NP28673 [19]. Data from the phase II NP28673 trial, indicate that alectinib determines an ORR of 50%, with a median PFS of 8.9 months in crizotinib-resistant ALK-positive NSCLC patients [18]. Based on these results, on 11 December 2015, the FDA approved alectinib to treat patients with advanced ALK-positive NSCLC, whose disease has worsened after, or who could not tolerate treatment with crizotinib. The randomized open-label phase III ALEX trial has compared alectinib with crizotinib in treatment-naïve ALK-positive NSCLC patients, demonstrated a better PFS and improved control of CNS disease, in patients treated with alectinib in first line setting [22].

Brigatinib is a novel ALK TKI that overcomes crizotinib resistance and shows great activity against the ALK TK gatekeeper mutations L1196M and G1202R [23]. The antitumor activity and safety profile of brigatinib was evaluated in a phase I/II trial, enrolling 79 ALK positive NSCLC patients [24]. Tumor response was observed in 100% of crizotinib naive patients, in 74% of crizotinib pre-treated patients and 83% of crizotinib-naive or crizotinib-pre-treated ALK-rearranged NSCLC with active, measurable, intracranial metastases. On April 28, 2017, the U.S. FDA granted accelerated approval to brigatinib for the treatment of patients with metastatic ALK-positive NSCLC who have progressed on or are intolerant to crizotinib. A randomized phase III trial ALTA-1, comparing in first line crizotinib versus brigatinib, is on-going.

Lorlatinib is a third-generation ATP-competitive selective ALK and ROS1 inhibitor, specifically designed and optimized to penetrate BBB. Preclinical data have shown that lorlatinib has activity against ALK resistant mutations, including G1202R, and regresses intracranial metastasis at doses much lower than the maximum tolerated dose [25]. Lorlatinib is ∼10-fold more potent against wild-type EML4-ALK and ∼40-fold more potent against EML4-ALK L1196M compared with crizotinib. Preliminary results from the phase I/II trial in 41 ALK-positive and 12 ROS1-positive NSCLC patients with or without CNS metastases showed an ORR of 50% [26]. In the patients evaluable for intracranial response, the ORR was 60%.

All these data indicate that resistance profiles may evolve over time and in response to sequential ALKis. The identification of the acquired resistance mechanisms at the molecular level will allow clinicians to personalize ALK-targeted strategies and to define the proper sequence of these drugs.

The temporal availability of crizotinib followed by second or third generation ALKis led to a majority of patients treated with this sequence, which we indicate with the term “classical group”; however more recently some patients started their ALK inhibition directly from a second generation ALK inhibitor, due to the opportunity of a clinical trial, leading to a different sequencing, which we call “experimental group”. In our Institute we retrospectively observed the outcomes of our ALK population, with the goal to answer the question whether the two strategies of sequencing are different in terms of efficacy, mainly progression free survival and intracranial activity.

## RESULTS

### Patients

Between 2012 and 2017 we treated 40 patients ALK-positive. Baseline demographic and clinical characteristics of the enrolled patients are summarized in Table 1.

**Table 1:** Baseline demographic and clinical characteristics of all treated patients

		Total (*n* = 40)
***n* (%)**
Gender		
	Female	22 (55.0)
	Male	18 (45.0)
Age, years		
	Median	54.2
	Range	28–86
Smoking status		
	Current-smoker	0 (0)
	Never smoker	32 (80.0)
	Former-smoker	8 (20.0)
PS (ECOG)		
	0	24 (60.0)
	1	11 (27.5)
	2	5 (12.5)
Histology		
	Adenocarcinoma	40 (100)
	Others	0 (0)
Stage at diagnosis		
	Locally-advanced	13 (32.5)
	Metastatic	27 (67.5)
Brain metastases at baseline		
	Yes	5 (12.5)
	No	35 (82.5)
Surgery^1^		
	Yes	7 (17.5)
	No	33 (82.5)
WBRT^2^		
	Yes	8 (20.0)
	No	32 (80.0)
Chemotherapy^3^		
	Yes	26 (65.0)
	No	14 (35.0)
Manteinance pemetrexed		
	Yes	7 (26.9)
	No	19 (73.1)
Chemotherapy cycles		
	Median	5.8
	Range	2–15

Legend: ^1^Lobectomy, pneumonectomy, metastasectomy. ^2^WBRT (Whole Brain Radiotherapy). ^3^Cisplatin, pemetrexed in most cases.

Patients were relatively young (median age, 54 years; range 28–86 years), with only 2 patients older than 70 years old, and most of them (80.0%) were non-smokers. There was a minimal prevalence of female sex (55.0%). Histology was adenocarcinoma for all the patients.

About Performance Status (PS) according to Eastern Cooperative Oncology Group (ECOG) scale at diagnosis, 24 patients (60.0%) had PS 0, 11 (27.5%) had PS 1, and 5 (12.5%) had PS 2. Thirteen patients (32.5%) presented with locally advanced, stage III, unresectable NSCLC and 27 (67.5%) with stage IV.

The classical group included 31 patients (77.5%), treated with crizotinib in the front line setting; 9 patients (22.5%) were included in the experimental group and received first-line second-generation ALKis (Table 2).

**Table 2:** Treatment groups

	Total (*n* = 40)
***n* (%)**
**Classical group^1^**	31 (77.5)
**Experimental group^2^**	9 (22.5)

^1^ First line with crizotinib.

^2^ First line with second-generation ALK inhibitor.

About the CNS involvement only 5 patients (12.5%) had baseline brain metastases (BM); 4 patients (12.9%) in the classical group, one (11.1%) in the experimental group.

Eight patients (20.0%) were treated with whole-brain radiation therapy (WBRT) before or in conjunction with ALKis, one of them also received later stereotactic radiosurgery (SRS). Platinum-based chemotherapy was administered before ALKis to 26 patients (65.0%), 7 also received pemetrexed maintenance therapy (Table 1).

In the crizotinib pretreated group, 29 patients out of 31 (93.5%) received subsequent ALKis treatment after first-line post progression (ceritinib in most cases, brigatinib, alectinib or lorlatinib); 9 patients out of 31 (29.0%) received an ALKis in third setting, 3 out of 31 (9.7%) also in fourth line (Table 3).

**Table 3:** Treatment lines with ALK inhibitors

	I line *n* (%)	II line *n* (%)	III line *n* (%)	IV line *n* (%)
**Group**				
Classical	31 (100)	29 (93.5)	9 (29.0)	3 (9.7)
Experimental	9 (100)	3 (33.3)	1 (11.1)	0 (0)

In the experimental group all the 9 patients (100%) were treated with ceritinib in the front line setting; only 3 (33.3%) developed a tumor progression at the time of analysis and received a further ALKis therapy (lorlatinib or brigatinib), one patient also received brigatinib as third line (Table 3).

### Efficacy

At the time of data cutoff, the median follow-up was 26 months for patients assigned to classical group and 15 months for experimental group.

**Figure 1:**
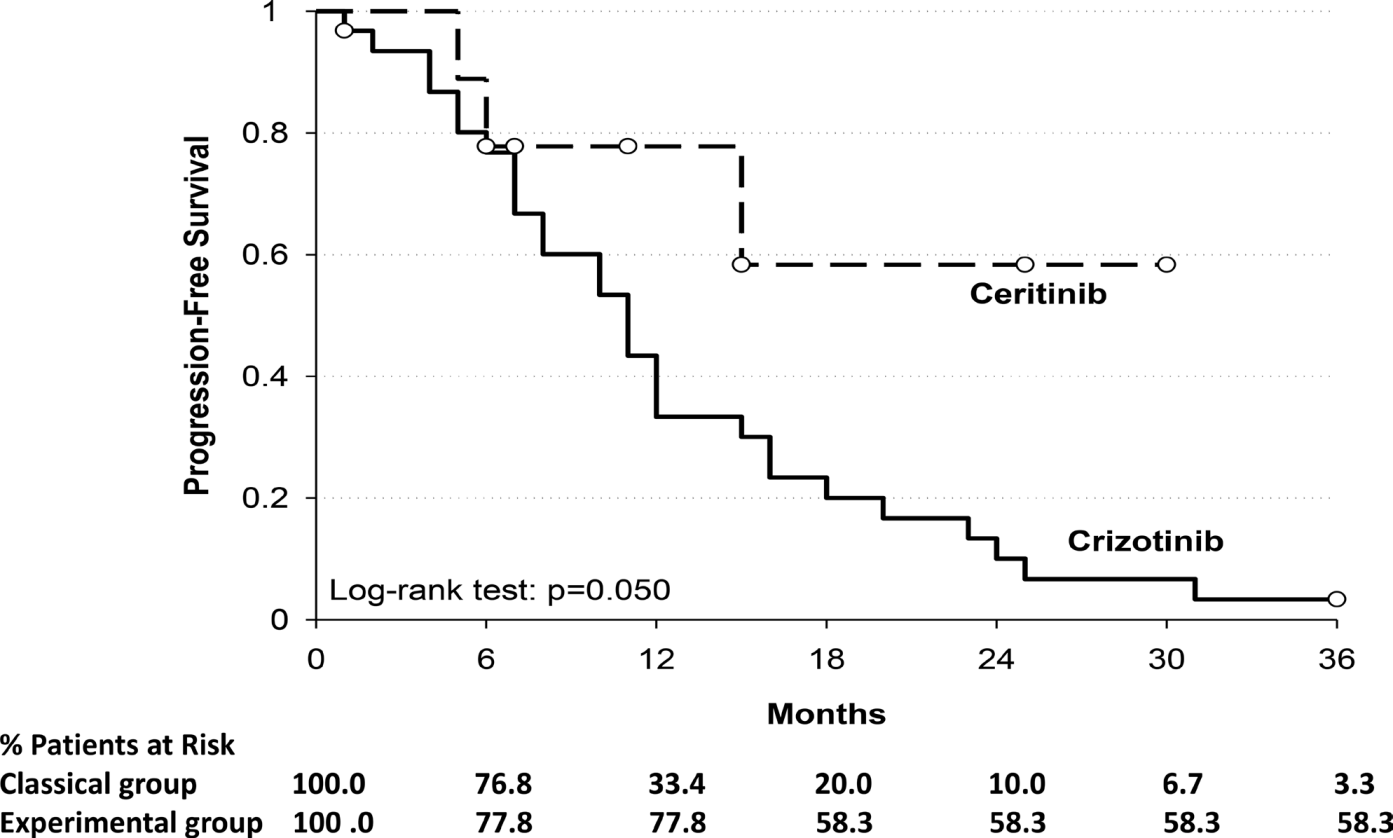
Shows the Kaplan–Meier curves for progression-free survival among patients who received crizotinib or ceritinib Y-axis: Probability of progression free survival; X-axis: time since treatment assignment (months). In the subgroup of patients of classical group (----) the median progression-free survival was 11 months; in the subgroup of patients of experimental group (__) the median progression-free survival was not reached.

The median PFS was 11 months for the classical group whereas it was not reached for experimental group (Figure 1); the 1-year PFS was 33.4% and 77.8% for classical and experimental group respectively (Table 4). This difference in PFS was confirmed by the Kaplan–Meier curves (*p* = 0.050).

**Table 4:** Progression free survival (PFS) for treatment groups

	PFS	
**Crizotinib**	**Ceritinib**
**Median**	11 months	---
**Time**		
6 months	76.8%	77.8%
12 months	33.4%	77.8%
18 months	20.0%	58.3%
24 months	10.0%	58.3%
30 months	6.7%	58.3%
36 months	3.3%	58.3%
	Log-rank test; *p* = 0.050.	

**Figure 2:**
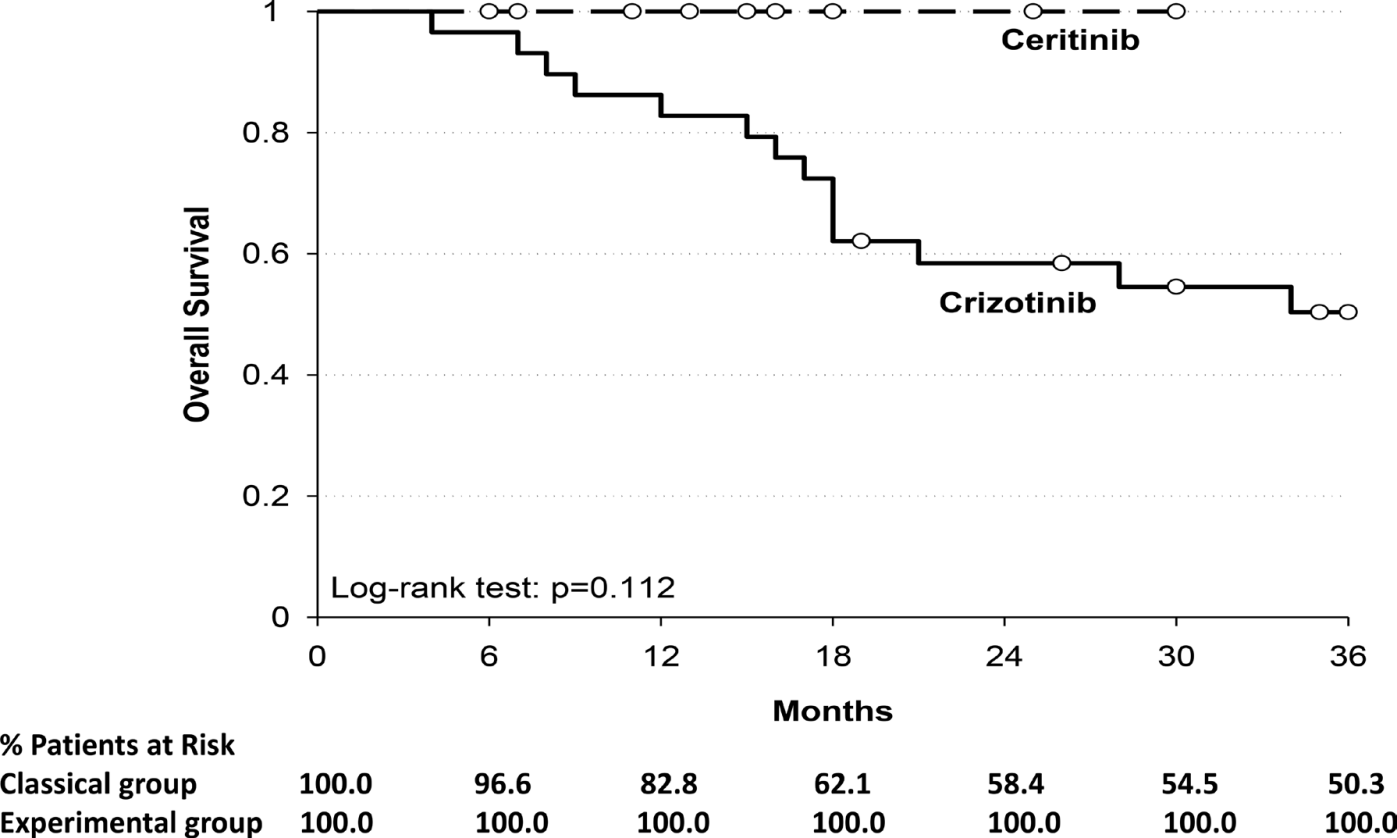
Shows the Kaplan–Meier curves for overall survival among patients who received crizotinib or ceritinib Y-axis: Probability of overall survival; X-axis: time since treatment assignment (months). In the subgroup of patients of classical group (---) the median overall survival was not reached; in the subgroup of patients of experimental group (__) the median overall survival was not reached.

The 1-year Overall Survival (OS) rate was 82.8% for classical group; all patients in the experimental group were alive after a median follow-up of 15 months (Table 5). The median OS was not reached for both groups. There was no statistically significant difference observed in OS (*p* = 0.112) (Figure 2).

**Table 5:** Overall survival (OS) for treatment groups

	OS	
**Crizotinib**	**Ceritinib**
**Median**	---	---
**Time**		
6 months	96.6%	100.0%
12 months	82.8%	100.0%
18 months	62.1%	100.0%
24 months	58.4%	100.0%
30 months	54.5%	100.0%
36 months	50.3%	100.0%
	Log-rank test; *p* = 0.112.	

The frequency of CNS involvement at baseline was the same in the two groups (*p* = 1,000). About the Cranial progression (C-PD), 10 patients (37.0%) of the 27 (87.1%) without brain metastasis in the classical group developed new intracranial lesions; median C-PD from crizotinib intake was 395 days (range 89–738 days). At the time of the analysis in the experimental group none of the eight patients (88.9%) without BM developed a cranial progression; however second-generation ALKis showed non-statistically significant improvement in C-PD (*p* = 0.07) (Table 6). As regards the five patients with brain metastases at diagnosis, 3 out of 4 in the classical group had a intracranial progression during crizotinib while the other patient had a stable disease after whole brain radiotherapy. Conversely, the only patient in the experimental arm with brain metastasis reached a complete response in the brain.

**Table 6:** Cranial progression (C-PD) for treatment groups

	Classical group	Experimental group
Brain metastases at baseline		
Yes *n* (%)	4 (12.9)	1 (11.1)
No *n* (%)	27 (87.1)	8 (88.9)
C-PD^1^		
Yes *n* (%)	10 (37.0)	0 (0)
No *n* (%)	17 (63.0)	8 (100)
Median	395	
Range	89–738	
Fisher Exact Test *p* = 0.07.		

^1^Cranial progression (development of cranial lesions) from the time of ALK inhibitors intake to the date of intracranial progression in patients without brain metastases.

## DISCUSSION

We evaluated the treatment and outcomes of 40 ALK-rearranged NSCLC patients treated in 5 years in a single Italian Institution; most of the patients have been treated into clinical trials or in expanded access programs, because in Italy crizotinib has been the only ALK inhibitor available up to now.

Given its monoinstitutional and retrospective nature, this analysis has some limitations. As a result, the number of patients in the two groups is imbalanced, those treated with crizotinib up front are more than those treated with a second generation ALK inhibitor as first-line; there is a difference also in the exposure time to several ALKis, availability of crizotinib has occurred before than the other ALKis, consequently patients started with crizotinib had the possibility to receive more other ALK-TKI.

However we think our analysis is an interesting picture of the “real-life” treatment of ALK-positive patients in a referral center, bringing to several suggestions.

Our patients are consistent with the ALK-positive epidemiology, quite young, never or light smokers, slightly more female than male.

Many patients received chemotherapy, mostly platinum-pemetrexed combinations, when crizotinib was not available in Italy; the patients-diagnosed in the last two years, did not receive it anymore, after demonstration by Profile 014 of the superiority of crizotinib over chemotherapy [4].

**Figure 3:**
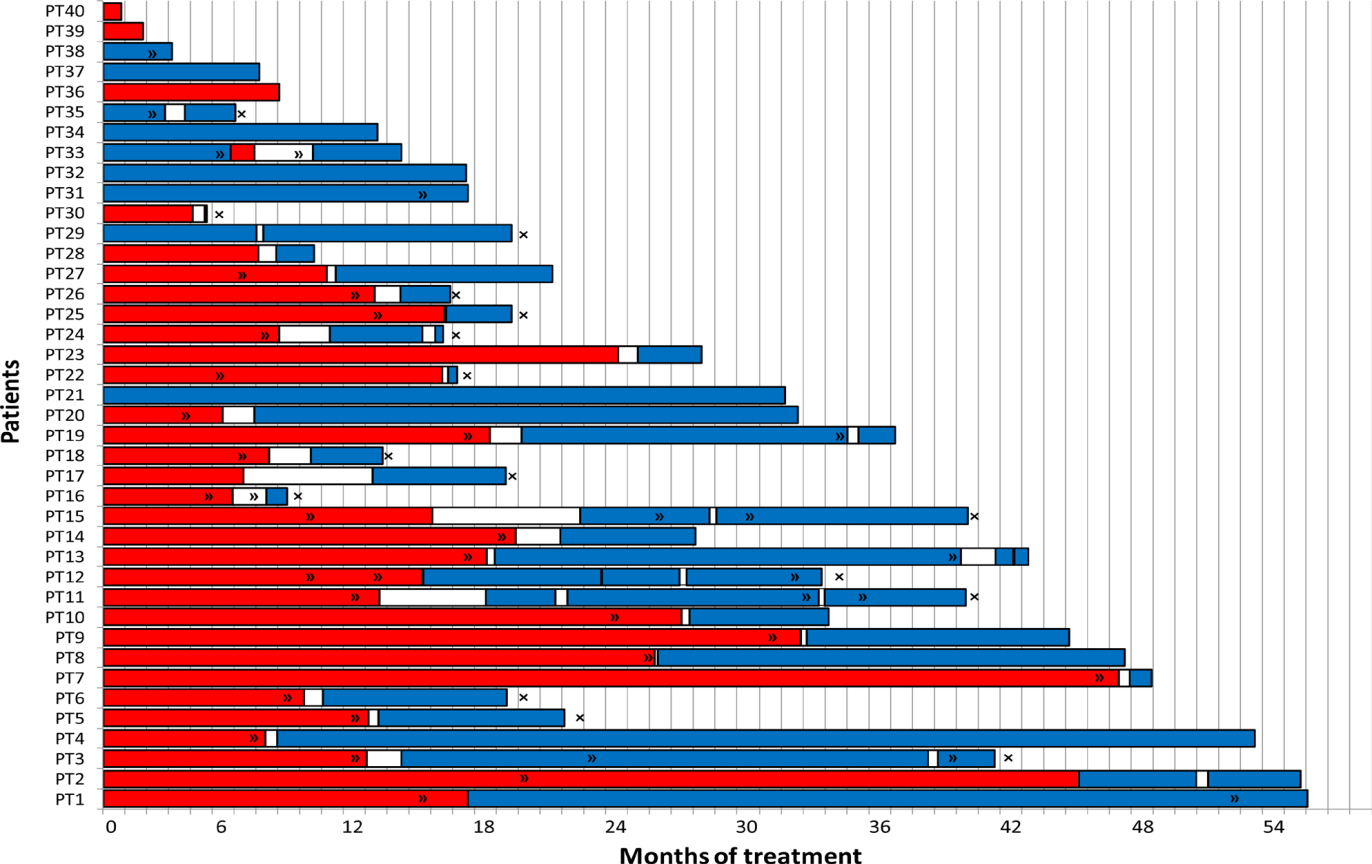
Shows treatment lines with ALK inhibitors X-axis: time since treatment assignment (months); red line: treatment with crizotinib; blue line: treatment with second/third generation ALK inhibitors; x: death; >>: disease progression. Y-axis: patients.

It is well clear that ALK-rearranged NSCLC tumors have many different available options of treatment with ALK-TKI, and Figure 3 shows how many patients had received several lines of ALKis, up to four. The pletora of available drugs explains the longer overall survival of ALK patients, much longer than the best survival in not-oncogene addicted patients, treated with chemotherapy. This is consistent with the findings by the Lung Cancer Consortium, where treatment with targeted agent in oncogene-driven lung cancer patients allows a longer overall survival [27]. The different ALK inhibitors have different potency, mutational coverage, PFS, ORR, CNS activity, allowing the strategy of sequential treatment. Overall, across the different ALKis, toxicities have been quite manageable and none had stopped treatment for ever for toxicity; overall quality of life was good, no G3–4 toxicities have occurred.

We do not know if starting with first generation crizotinib as first ALK-inhibitor is different from starting with second/third generation-ALKis in terms of outcomes, progression free survival and overall survival; primary treatment with 2nd-generation ALK inhibitors may potentially delay or prevent resistance development and reduce CNS involvement.

Our analysis has revealed that the experimental approach has been associated with a significant improvement in progression free survival. Probably due to the small number of patients enrolled and the different time and lines of exposure to ALK inhibition the overall survival does not differ significantly, however it seems there is a positive trend for the experimental group; it should be also noted that the median survival had not been reached in either group and that in the experimental group there have been no deaths.

Intracranial progression has been defined as the development of brain metastases. Despite the relative small and unbalanced sample size, the majority of patients of both groups (27 in the classical arm and 8 in the experimental arm) had no brain metastases at baseline. Moreover, we even investigated this endpoint as the overall population had been participating in heterogeneous studies (with some of them being not so recent), in which the evaluation of the central nervous system response had been methodologically different.

Since ALK population tends to develop brain metastasis quite often, and knowing that crizotinib levels in the brain are less than in the blood or in other tissues [11], we expect a less amount of CNS metastases in the experimental group.

Although the experimental group is much lower than the classical group and the median follow-up is shorter, however we observe less new brain metastasis than in the classical group. Actually in the experimental group none of the patients without brain metastases developed them during their treatment and at tumor progression CNS has never been involved.

Although the endpoint considered was not statistically significant, there was a trend in favour of the experimental group, which may suggest that the intracranial disease control is overall better with second generation ALKis, in line with the results of recent prospective trials [15, 22, 28].

In our analysis we have no data on *EML4-ALK* variants, all patients had a central tissue revision, confirming the ALK positivity, with the method provided by the trial, fluorescence in-situ hybridization (FISH) or immunohistochemistry (IHC). Few retrospective studies [29, 30] have focused on specific *EML4-ALK* variants and clinical responses to ALK inhibitors. It might be possible that the fusion variants could play a role in explaining the response and its duration. Further multicenter, prospective studies are needed to investigate the association between stratification of patients by variant-specific genotype and clinical responses to ALKis.

It could be also of interest to know if the percentage of ALK-positive cells might correlate with the response of ALKis, as demonstrated in few retrospective studies [31]. Again, we do not have these data, because of central tissue revision.

Currently the best treatment strategy for ALK positive patients has to be determined yet. The ALEX trial has recently demonstrated a significant improvement in progression free survival and control of CNS disease, in patients treated with alectinib versus crizotinib in first line setting [22].

Other prospective trials are on-going comparing crizotinib-first generation treatment versus second/third generation treatment as first-line in ALK patients; those trials should answer the question whether the classical and the experimental approach bring to different outcomes.

As we have described above, our analysis is a monoinstitutional and retrospective study. Despite the small sample size, the study has revealed the better outcome of patients treated with second generation ALK inhibitors from the beginning, with a statistically significant improvement in progression-free survival.

Likely due to the small size of the groups, the different follow-up in the two arms, the availability of clinical trials with new ALK inhibitors at different times, statistically differences in other endpoints, namely overall survival and brain progression, have not been found.

Despite these limitations, we think the casistic could be of interest. In the absence of an intelligent way to guide the therapeutic sequence and on the basis of the availability of ALKis, this retrospective analysis may give a real picture of the outcome of ALK positive lung cancer patients.

Furthermore our analysis seems to suggest that survival tends to be better and the brain metastasis tend to onset much later when starting with new ALKis; similarly to the recent published prospective trials where second generation ALKis have demonstrated better systemic and intracranial disease control [15, 22, 28].

We think this retrospective analysis may give some hints about the preference between different sequential strategies, the first and second/third generation of ALKis. With new ALK inhibitors on the horizon, the sequencing of these drugs is a topic of further research.

## MATERIALS AND METHODS

Patients were 18 years of age or older, with an ECOG performance status of 0 to 2, measurable disease (according to Response Evaluation Criteria in Solid Tumors [RECIST], version 1.1), and adequate hepatic, renal, and bone marrow function Patients with asymptomatic brain or leptomeningeal metastases were eligible; previous CNS radiotherapy was allowed if completed at least 14 days before enrollment. They had ALK-positive (as assessed locally by immunohistochemistry and/or fluorescence *in situ* hybridization), locally advanced, recurrent, or metastatic non-squamous NSCLC, treatment-naive or after chemotherapy; provided that ≥ 3 weeks had elapsed since last cytotoxic treatment. Prior radiotherapy as well as presence of brain metastases were not exclusion criteria. Patients with corrected QT (QTcF) > 470 ms using Fridericia’s correction on screening ECG were excluded. All the patients signed an informed consent, the drugs being assigned into a clinical trial or in a compassionate use, with a study-specific consent.

All the patients underwent tumor imaging at baseline, including scans of the brain. Subsequent tumor evaluation, including systematic brain imaging in all patients, was performed every 6 weeks until disease progression. Tumor response was assessed with the use of RECIST, version 1.1.

We have assessed OS, PFS, ORR and C-PD in two different treatment groups: patients in the classical group treated with first-line crizotinib therapy and patients in the experimental group treated with first-line next-generation ALKis.

OS was determined from the first date of ALKis intake to the date of death from any cause, or the last survival follow-up. PFS was measured from the first day of ALK-is intake to the documented progression, or death from any cause. Tumor response was assessed as complete response (CR), partial response (PR), stable disease (SD), or progressive disease (PD), in accordance with the standard RECIST (version 1.1). The ORR was defined as CR plus PR.

Cranial progression was determined from the time of ALKis intake to the date of intracranial PD (development of new intracranial lesions) in patients without brain metastasis.

The Kaplan–Meier method was used to estimate probabilities of time-to-event end-points; treatment groups were compared using a two-sided log-rank test. The Fisher’s exact test was used for comparing the C-PD between the two different treatment groups. *P <* 0.05 was considered statistically significant.
